# Identifying psychiatric diagnosis from missing mood data through the use of log-signature features

**DOI:** 10.1371/journal.pone.0276821

**Published:** 2022-11-17

**Authors:** Yue Wu, Guy M. Goodwin, Terry Lyons, Kate E. A. Saunders

**Affiliations:** 1 Mathematical Institute, University of Oxford, Oxford, United States of America; 2 Alan Turing Institute, London, United Kingdom; 3 Department of Mathematics and Statistics, University of Strathclyde, Glasgow, United Kingdom; 4 Department of Psychiatry, University of Oxford, Oxford, United Kingdom; 5 Oxford Health NHS Foundation Trust, Warneford Hospital, Oxford, United Kingdom; 6 NIHR Oxford Health Biomedical Research Centre, Oxford, United Kingdom; National Institutes of Health, UNITED STATES

## Abstract

The availability of mobile technologies has enabled the efficient collection of prospective longitudinal, ecologically valid self-reported clinical questionnaires from people with psychiatric diagnoses. These data streams have potential for improving the efficiency and accuracy of psychiatric diagnosis as well predicting future mood states enabling earlier intervention. However, missing responses are common in such datasets and there is little consensus as to how these should be dealt with in practice. In this study, the missing-response-incorporated log-signature method achieves roughly 74.8% correct diagnosis, with f1 scores for three diagnostic groups 66% (bipolar disorder), 83% (healthy control) and 75% (borderline personality disorder) respectively. This was superior to the naive model which excluded missing data and advanced models which implemented different imputation approaches, namely, k-nearest neighbours (KNN), probabilistic principal components analysis (PPCA) and random forest-based multiple imputation by chained equations (rfMICE). The log-signature method provided an effective approach to the analysis of prospectively collected mood data where missing data was common and should be considered as an approach in other similar datasets. Because of treating missing responses as a signal, its superiority also highlights that missing data conveys valuable clinical information.

## Introduction

The rapid emergence of mobile technologies has transformed the way in which mental health data can be collected. Until recently clinicians were wholly reliant on anamnestic approaches and were hampered by the inaccuracy of retrospective recall regarding psychiatric symptoms. Mobile technologies have enabled the efficient capture of self-reported symptoms in an ecologically valid and prospective manner. A number of different approaches to the analysis of longitudinal mood data have been employed [1–3]. However missing data is ubiquitous and poses a significant methodological challenge. Mood data may be missing unrelated to mood state or in fact be a consequence of current mood state. Such missingness could be considered as a complex status of the three missingness mechanisms defined in [4], namely, missing completely at random (MCAR), missing at random (MAR), and missing not at random (MNAR). Standard approaches such as mean imputation may inadvertently lead to the loss of important information [5].

We therefore proposed a missing-response-incorporated log-signature-feature-based (MRLSF) machine learning model which encodes missing values to a signal. The real challenge of incorporating missing data as a channel is that the resulting data stream is asynchronous. That is to say, events in different channels happen at different times. In particular, one does not get mood data and the omission of mood data happening at the same time. Rough path theory and (log-)signatures provide a robust theoretically justifiable framework for analysing multi-dimensional asynchronous streamed data [6]. By pipe-lining these two processes: a) recording the omission of data as a new channel, b) the signature approach to analysing the resulting asynchronous data, we establish a novel and moderately generic approach to handling missing data and demonstrate its value for the analysis of the mood data.

In a previous analysis we demonstrated that a signature-feature model could be successfully applied to 6-dimensional self-reported mood data [1], however missing data was excluded for analysis. In this study, we used this missing-response-incorporated log-signature-feature-based machine learning model to re-analyse weekly mood data collected from the AMoSS study [7] which used self-reported mood data and wearables to distinguish between individuals with bipolar disorder (BD), borderline personality disorder (BPD) and healthy controls (HC). We sought to test whether this new analytic approach was superior to a standard approach to mood quantification, which adopts the mean metric without considering missing values [7], in its ability to distinguish these diagnostic groups. The performance was further compared to various commonly-used imputation methods: k-nearest neighbors (KNN) [8], probabilistic principal components analysis (PPCA) [9, 10] and random forest-based multiple imputation by chained equations (rfMICE) [11, 12].

## Methods

### Data

Participants with BD or BPD and healthy volunteers reported their mood and health using Altman Self-Rating Mania Scale (ASRM) [13], the Quick Inventory of Depressive Symptoms (QIDS-SR16 or QIDS for short) [14], EQ-5D (EuroQoL) and the Generalised Anxiety Disorder Assessment (GAD-7) [15]. ASRM is a short, five-item self-assessment questionnaire assessing the presence and severity of manic or hypomanic symptoms. A score of ASRM above 5 is claimed to indicate a manic episode [13]. QIDS-SR16 contains 16 items covering the nine DSM-IV symptom criterion domains [16] with the total score ranging from 0 to 27. A score of QIDS above 10 indicates moderate or very severe depression. EQ-5D is a standardised validated instrument assessing mental health status, and only the item where participants quantify their quality of life (0–100%) was used. The reported population mean in the UK is 82.8 [17]. GAD-7 contains seven items which measure severity of various signs of GAD, with the total score ranging from 0 to 21. A score of GAD-7 above 10 indicates moderate or severe anxiety. These four questionnaires allow one to track participant’s mood and health over time.

ASRM, QIDS, EQ-5D and GAD-7 data were collected from 142 individuals as part of the AMoSS study [7] and the participants completed standardised questionnaires on a weekly basis using the True Colors mood monitoring system [18] after receiving a text or email prompt. Two of the 142 participants either withdrew consent or had no clinical diagnosis and were therefore excluded from analysis. We further excluded one participant who failed to provide at least ten weeks data as part of the analysis is based on information in data of at least ten weeks. Of the remaining 139 participants, 53 were diagnosed as bipolar disorder and 34 were borderline personality disorder. The demographic details of the participants are summarised in Table 1. The four different types of data were aligned based on calendar weeks during per participant’s entire study. The duration of one participant’s entire study is defined as the time period of their task-active weeks. All identical duplicate values were checked and removed, and only the first response of a week was kept if multiple responds happened within that week. Each participant was associated with a stream of four-dimensional scores for ASRM, QIDS, EQ-5D and GAD-7. A score of ‘-1’ represents a missing response.

**Table 1:** Demographic characteristics of the three groups (the appropriate distributions are summarised in the form of the median + /− in the interquartile range).

Group	Recruited	For analysis	Weeks in study	Ages	Gender(males)
*BD*	54	53	52±12	38±20	19
*HC*	52	52	52±2	37±20	19
*BPD*	34	34	52±1	34±13	3

**Recruited**: The number of participants in each of the three groups who participated in the study without withdrawing consent or having no clinical diagnosis;

**For analysis**: The number of participants in each of the three groups who have been identified as recruited and also provided at least two weeks data

For each participant we computed the mean of their weekly scores for the mood vector [ASRM, QIDS, EQ-5D, GAD-7]. We can associate with any collection of participants a covariance matrix reflecting the correlations of the moods. We computed these correlations for each diagnostic group and compared them. In the following matrix, each cell contains correlations between outcomes of two tests, listed for BD, HC and BPD sub-populations. Note different diagnostic groups give different pairwise correlations.

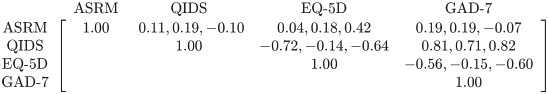


**Fig 1:**
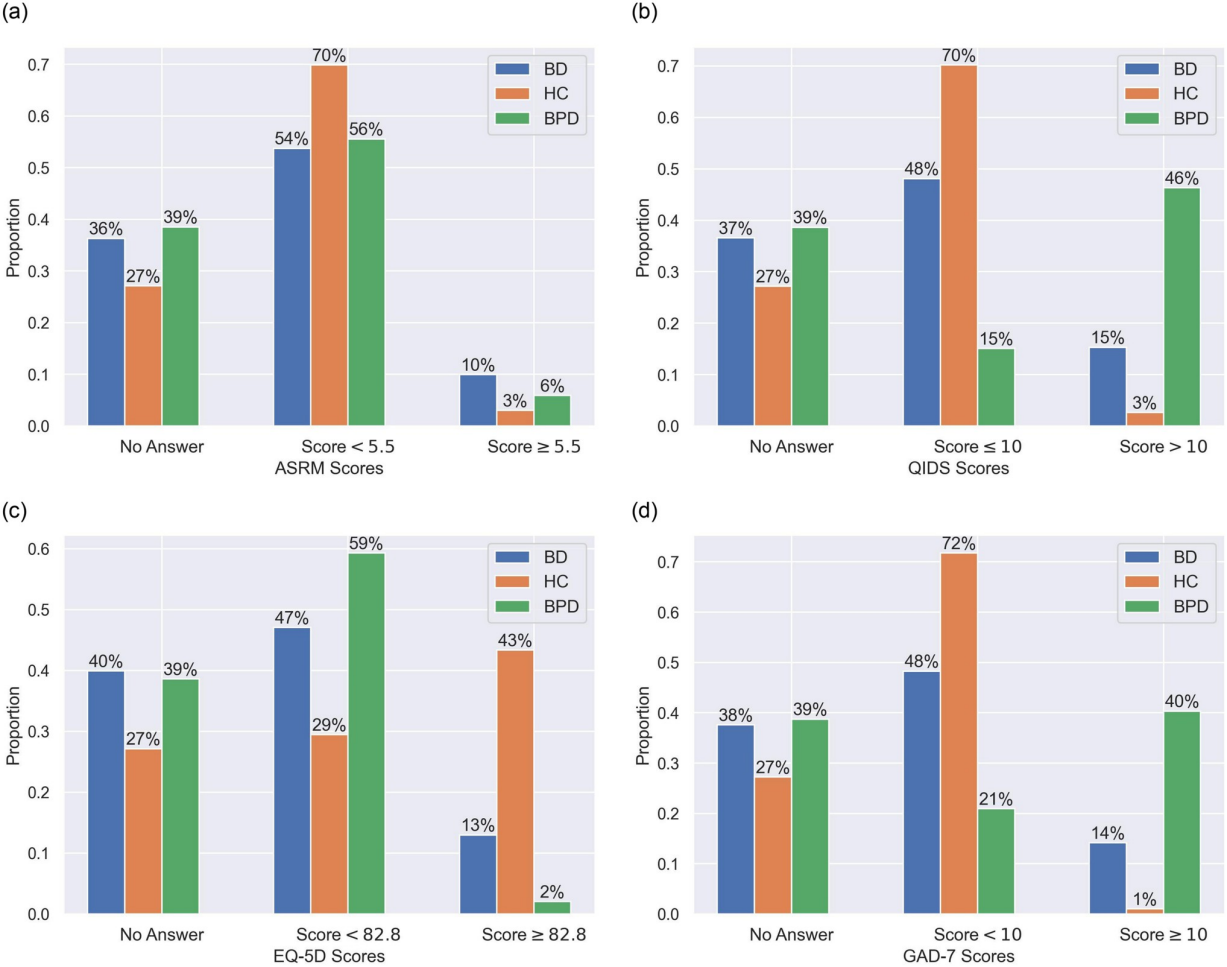
Bar charts: The proportion of time each participant group spent in the respective clinical states for each questionnaire (ASRM, QIDS, EQ-5D and GAD-7), where the total numbers of weeks for BD/HC/BPD are 3143/2816/1991. (a) ASRM. (b) QIDS. (c) EQ-5D. (d) GAD-7.

We had two ways of summarising the data streams and investigating the prevalence of missing responses in different diagnosis groups. Looking at one of ASRM, QIDS, EQ-5D and GAD-7 and one of the diagnostic groups, we can ask what percentage of the group failed to complete the assessment, what percentage of the group got a score below the cutoff, what percentage of the group got a score above the cutoff. This data is presented in Fig 1. One notes both BD and BPD patients were more likely to have missing responses.

**Fig 2:**
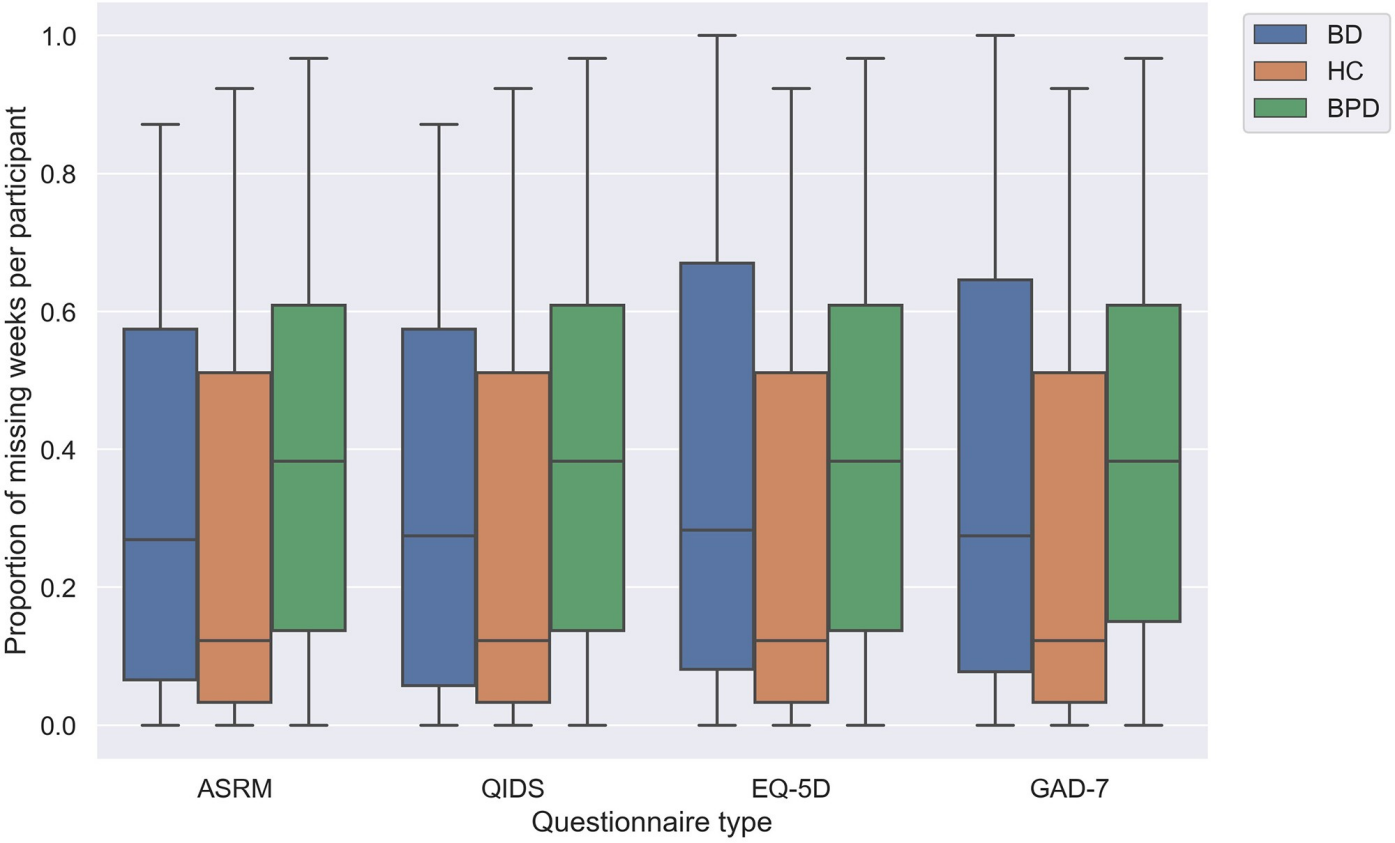
Boxplot: The proportion of missing responses per participant (median ± the interquartile range) in each of three diagnosis groups.

Furthermore, for each participant we calculated the proportion of weeks giving missing responses per type of questionnarie over the period of the study. Within each diagnostic group, we computed and plotted the medians (± the interquartile range) as in Fig 2. Consistent with Fig 1, HC had clearly the lowest median values for the number of unreturned questionnaires and BPD, on the contrary, had the highest median values.

#### Ten-week windows

To make the most of the small dataset, we split each participant’s mood data into a sequence of ten-week windows, and analysed this collection of ten-week data streams. This generated 6690 four dimensional streams with ten-week data drawn from 139 participants. If instead using 20-week observations as described in [1], we would have to exclude 13 of 140 participants whose duration is less than 20 weeks. One consequence of this approach is that the mood sequences captured in the different streams maybe highly correlated since there will be many windows from any individual. For this reason, the validation of our analysis needs to be done with care. Because of this we used k-fold cross-validation such that each individual was in the hold-out set once and the model was retrained without them. We then tested the model on this individual’s windowed data.

### Ethic statement

The study protocol was approved by the NRES Committee East of England—Norfolk (13/EE/0288) and all participants gave written informed consent.

### Features extraction

#### Log-signature features

In recent year, signatures of continuous paths generated from longitudinal data is considered as an efficient feature set for learning purpose because of its nature to capture the order in which events occur and the nonlinear effect of the evolving systems [19]. So far, the signature method has significantly contributed to automated recognition of Chinese handwriting [20, 21], formulation of appropriate stochastic partial differential equations to model randomly evolving interfaces [22, 23], skeleton-based human action recognition [21, 24, 25], diagnosis of Alzheimer’s disease [26] and speech emotion recognition [27, 28]. Some of them utilised log-signature features instead of signature ones to benefit from dimension reduction, where the log-signature of a path is indeed the logarithm of its signature.

#### Signatures: The definition

Consider $R^{d}$-valued time-dependent, piecewise-differentiable paths of finite length. Such a path *X* mapping from time domain [*a*, *b*] to $R^{d}$ is denoted as $X:\left[a,b\right]\to R^{d}$. For short we will use *X*_*t*_ for *X*(*t*), *t* ∈ [*a*, *b*]. Each coordinate path of *X* is a real-valued path and denoted as *X*^*i*^, *i* ∈ [*d*] with [*d*] ≔ {1, …, *d*}. The *signature* of a path $X:\left[a,b\right]\to R^{d}$, denoted by *S*(*X*)_*a*,*b*_, is the infinite collection of all iterated integrals of *X*. That is,

$$\begin{array}{c} S{\left(X\right)}_{a,b}≔\left(1,S{\left(X\right)}_{a,b}^{1},\ldots ,S{\left(X\right)}_{a,b}^{d},S{\left(X\right)}_{a,b}^{1,1},S{\left(X\right)}_{a,b}^{1,2},\ldots \right), \end{array}$$ (1)

where, the first term is 1 by convention, and the superscripts of the terms after the first term run along the set of all multi-index {(*i*_1_, …, *i*_*k*_)|*k* ≥ 1, *i*_1_, …, *i*_*k*_ ∈ [*d*]} with the coordinate iterated integral being

$$\begin{array}{c} S{\left(X\right)}_{a,b}^{i_{1},\ldots ,i_{k}}≔\int_{a<t_{k}<b}\ldots \int_{a<t_{1}<t_{2}}\text{d}X_{t_{1}}^{i_{1}}\ldots \text{d}X_{t_{k}}^{i_{k}}. \end{array}$$ (2)

The finite collection of all terms $S{\left(X\right)}_{a,b}^{i_{1},\ldots ,i_{k}}$ with the multi-index of fixed length *k* is termed as the *kth level of the signature*. The truncated signature up to the *p*th level is denoted by ⌊*S*(*X*)_*a*,*b*_⌋_*p*_. In machine learning context, truncated signature features are always obtained by truncating the original signature to some finite level.

#### Signatures as a natural feature set

For a path of finite length, the corresponding signature is the fundamental and faithful representation that ensures that the *incremental* effects of the path can be locally approximated by linear combinations of signature elements and any functionals on the path can be rewritten as a function on the signature (also known as universality of the signature). Moreover, the signature feature is able to deal with data streams of various length and unequal time spacing by its nature. Reparameterising a path does not change its signature, which allows signature features remain the same regardless of different sampling rates of data streams or time series.

#### Log-signatures

The log-signature of a path is defined as the logarithm of the signature of the path X, i.e., log(*S*(*X*)), denoted by *lS*(*X*). Because the logarithmic map is bijective, there is a one-to-one correspondence between the signature and the log-signature. The big advantage of logarithmic signatures compared to signatures is that they further reduce the dimension of the input while preserving most of signature properties. Note that the log-signature does not have universality as the signature, and thus it needs be combined with non-linear models for learning task.

#### Log-signatures from discrete data

For a discrete data stream **x** = (**x**_1_, …, **x**_*n*_), where **x** contains *n* observations, and the *i*th observation **x**_*i*_, *i* ∈ [*n*], is assumed to be a *d*-dimensional column vector at the *i*th time point, one needs to convert it to a $R^{d}$-valued path of finite length via piecewise linear interpolation or other transforms in order to compute log-signature. The availability of Python packages *iisignature* [29] and *esig* allows easy calculation of log-signature, where the linear interpolation is implemented automatically by the packages.

#### Encoding missing data

Among all the 139 valid participants in our study, 90% missed a response on a least one occasion during their task-active weeks. Log-signature features allow missing responses to be included in the analysis without the need for imputation. To achieve this, the missing events are translated into a new counting process [4] in an accumulative manner. An example is illustrated below for the procedure.

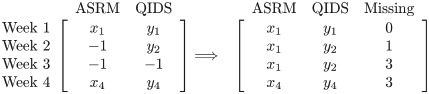


The left block contains 2 dimensional data of four consecutive observations, where -1 represents one missing observation; in the right block all missing places are filled with valid values that happened in the corresponding nearest past, while an additional dimension is added to count missing events cumulatively at each time points.

In the general case, if one works on data with N many time points, the accumulative missing counts can be generated for each of the N time points by calculating the sum of missing observations up to that particular time point; meanwhile each missing observation, i.e., input “-1” in our case, is replaced by the valid value that happened in the nearest past, which is referred as the *feed forward* method. This does not imply that the missing responses are assumed to take the same value as their nearest valid responses. By doing this, the increments in both observation and missing counts can be preserved and captured, which are indeed the most critical characteristic in the log-signature method together with their functionals [19].

After transforming missing responses, one then normalises and accumulates the data like in [3] to make it scale-free in order to apply log-signature transformation. Note that the description above can be applied to signature features.

### The workflow

**Fig 3:**
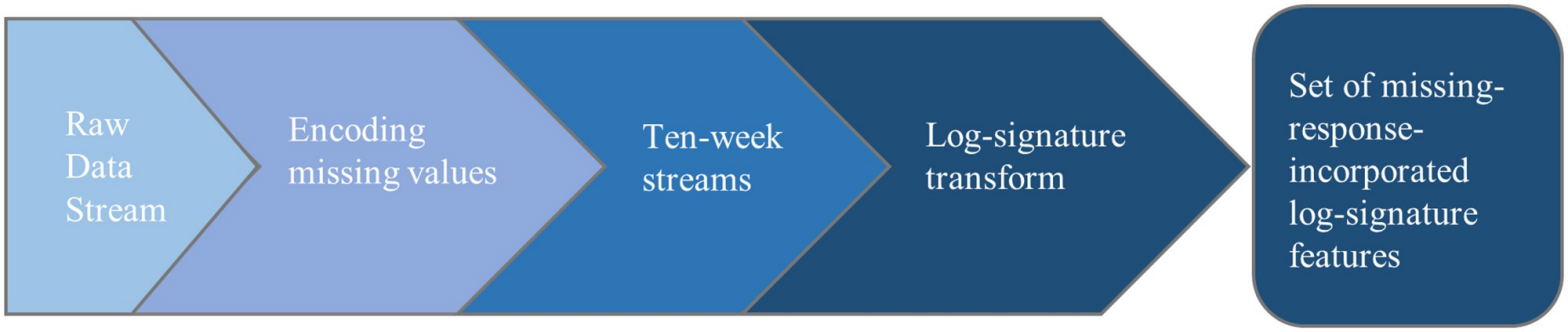
The workflow of feature extraction.

For our purpose, we extracted the consecutive concatenated observations for each participant, incorporated the missing data, divided it into ten-week streams and then calculated the corresponding log-signature features via Python package iisignature, where the log-signature features were truncated to level 3. To distinguish from standard log-signature features, our features were named the missing-response-incorporated log-signature features (MRLSF). The workflow can be found in Fig 3.

### Signature-based classification

In order to investigate the role of ASRM, QIDS, EQ-5D and GAD-7 scores in differentiating between healthy controls and different patient groups, a missing-response-incorporated log-signature-based classification model (MRLSM) was developed to classify the diagnostic group a participant belonged to. We conducted a 3-fold cross-validation on participant level. For each of 139 participants, all streams of 10 consecutive concatenated observations, no matter missing or not, was collected and transformed to MRLSF for this task, with their labels the same as the diagnostic group of this particular participant. Note that there are no cross-over between the streamed data of participants in the train set and the ones in the corresponding hold-out set. The proposed model was based on a random forest classifier and was trained on the input-output pairs, i.e., MRLSF and their labels, of each training set and predicted class probabilities on MRLSF from the hold-out set. As a by product, ten most significant variables of MRLSF were identified.

#### Participant-level classification

Note that the predicted probabilities and therefore the predicted labels obtained above are for ten-week data streams. Both hard and soft voting [30–32] were applied to obtain predicted labels for each participant. In a hard voting, also known as majority voting, the majority wins. The soft voting predicts a label based on the largest predicted value of the sum of the predicted probabilities.

#### Comparison models

For comparison, we attempted a naive method which was justified by clinic practice and several state-of-the-art imputation methods. For the naive method, a random forest classifier was trained on features extracted through a clinic-used metric based on the average score in each category over the valid scores in ten consecutive observations. We assessed three different imputation methods: K-nearest neighbors (KNN), probabilistic principal component analysis (PPCA) and randon-forest-based-multiple imputations by chained equations (rfMICE), where the last two have been used and compared in healthcare research [10]. The mechanics of three imputations are different: KNN defines a set of K-nearest neighbors for each weekly observation and then replaces the missing response for a given variable by averaging non-missing values of its neighbors; PPCA as a variant of vanilla PCA, estimates missing data on an expectation-maximization algorithm [33]; MICE creats multiple imputations for multivariate missing data through an iterative algorithm based on chained equations which utilises an imputation model specified separately for each variable and involves the other variables as predictors. For these imputation methods, we imputed missing responses first, extracted all the four-dimensional ten-week streams for each participant and trained a random forest classifier on the flattened vectors of data streams. The performance of MRLSM at level 3 and the ones from comparison models for classifying the diagnostic groups were measured in terms of accuracy. Meanwhile the confusion matrices of methods were generated to allow more detailed analysis, from which f1 scores for different diagnostic groups were computed. To assess the separation ability of different methods, we created the receiver operating characteristic curves (ROC) at various threshold settings and computed areas under curve (auc). Separately, we examined the raw data of these patients who were only identified by MRLSM.

#### Spectrum analysis

To further test the performance of the MRLSM (level 3), we investigated the likelihood of each of the three groups being categorised into the correct group. The probability vector of each participant being classified into each group was calculated and then projected onto the equilateral triangle, with each vertex representing one of the three groups. For example, if the inferred probabilities of one participant being classified as BD, HC and BPD are 0.1, 0.5 and 0.4 respectively, then the corresponding probability vector is [0.1, 0.5, 0.4]. This vector is indeed on a 3-dimensional triangle surface [*p*, *q*, 1 − *p* − *q*], with non-negative *p*, *q* and *p* + *q* ≤ 1. This triangle is the equilateral triangle that all the inferred 3-dimensional probability vectors will be sitting on. In order to demonstrate group-dependent characteristics, the probability vectors of patients from the same group were visualised in the same 3-dimensional equilateral triangle surface.

#### Summary

We used the publicly available Python iisignature package (version 0.23) to calculate log-signatures of data streams, Python numpy package (version 1.19.0) for data manipulations and processing, Python scikit-learn package (version 0.24.0) for KNN imputation, machine learning tasks and matplotlib for plotting and graphics (version 3.2.1). For PPCA and rfMICE imputation, we relied on pca-magic package (https://github.com/allentran/pca-magic) and miceforest (version 2.0.3) respectively.

The study was approved by the NRES Committee East of England—Norfolk (13/EE/0288).

A summary of models can be found in Table 2.

**Table 2:** A summary of models, where MR is short for missing responses, RF short for random forest.

Task	Base model	Raw data length	Model	Feature extraction	
				MR integration	Signatures
Classification	RF classifer	10	MRLSCM (level 3)	Yes	Yes
			Naive model	No	No
			KNN model	Yes	No
			PPCA model	Yes	No
			rfMICE model	Yes	No

## Results

### Classification of the diagnostic group

Under majority voting, MRLSM (level 3) categorised 74.8% of participants into the correct class while the naive model only classified 64.0% of participants correctly. The accuracies from KNN, PPCA and rfMICE were 70.5%, 68.3% and 67.0% respectively. Accuracies of the performance under soft voting can be found in Table 3. The accuracy from MRLSM improved with transformation of missing responses, indicating that missing responses bring additional information and therefore enhance the performance of the model.

**Table 3:** Accuracies for group classification under hard and soft voting schemes using different models.

Voting scheme	MRLSM	Naive model	KNN	PCCA	rfMICE
Hard	74.8%	64.0%	70.5%	68.3%	67.0%
Soft	72.7%	62.5%	69.8%	67.6%	67.2%

**Fig 4:**
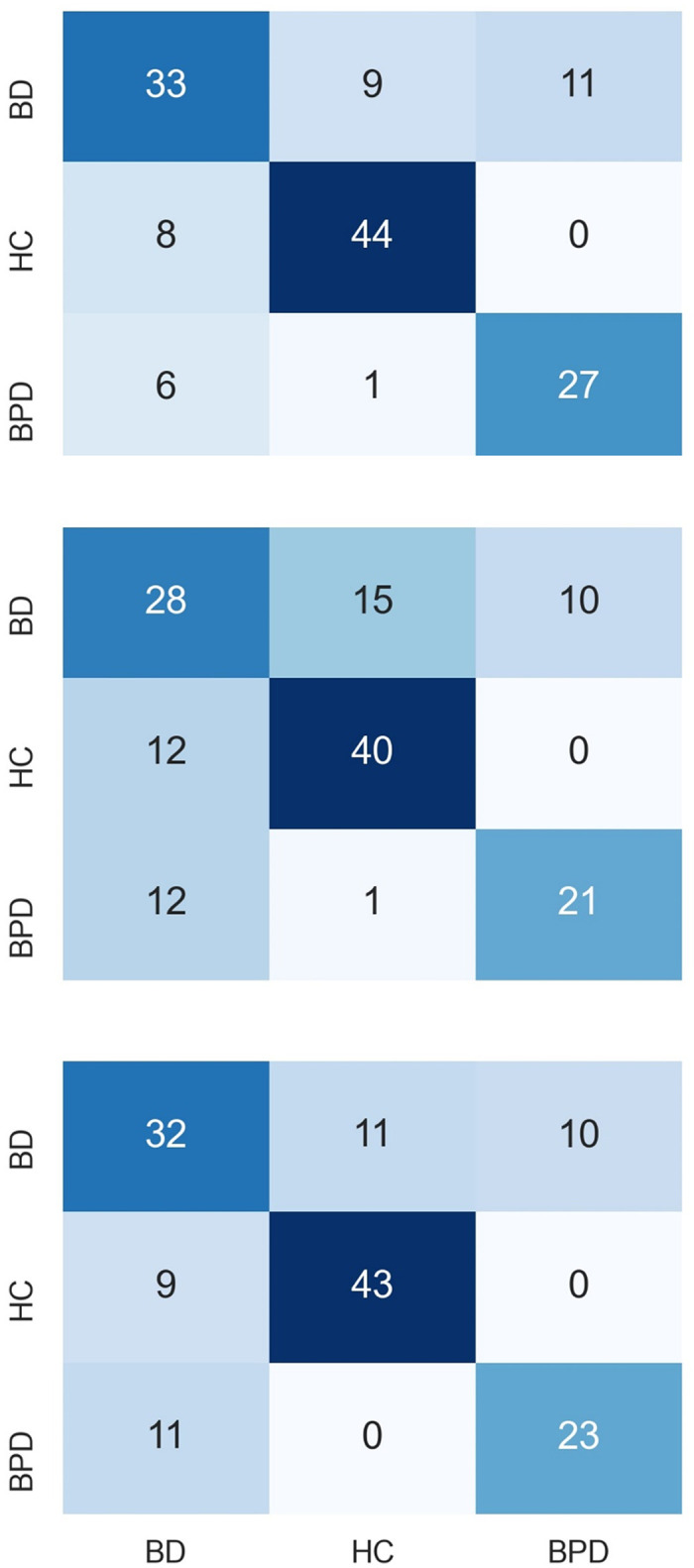
Confusion matrices of MRLSM, the naive model and KNN model. Upper: MRLSM. Middle: the naive model. Bottom: KNN.

We also output confusion matrices from different models, which illustrated the detailed correct and false classification for each group and allowed for computing f1 scores in Table 4. Table 4 shows that the MRLSM had the highest f1 score in all three classes. All models achieved their lowest f1 scores for classifying BD. However, by encoding the missing information into the model, the ability of classifying BPD was significantly enhanced by 24% from 0.533 (the naive model) to 0.660 (MRLSM). Note that all imputation-based models were superior to the naive model in recognising bipolar patients. Among imputation methods, KNN achieved the best performance. For further comparision, we presented confusion matrices from MRLSM, the naive model and KNN in Fig 4.

**Table 4:** F1 scores for group classification under hard and soft voting schemes using different models.

Model	BD		HC		BPD	
	Hard	Soft	Hard	Soft	Hard	Soft
**MRLSM**	0.660	0.634	0.830	0.822	0.750	0.714
**Naive model**	0.533	0.514	0.741	0.741	0.646	0.615
**KNN**	0.610	0.604	0.811	0.807	0.687	0.676
**PPCA**	0.580	0.574	0.792	0.811	0.667	0.620
**rfMICE**	0.603	0.602	0.784	0.796	0.600	0.613

**Fig 5:**
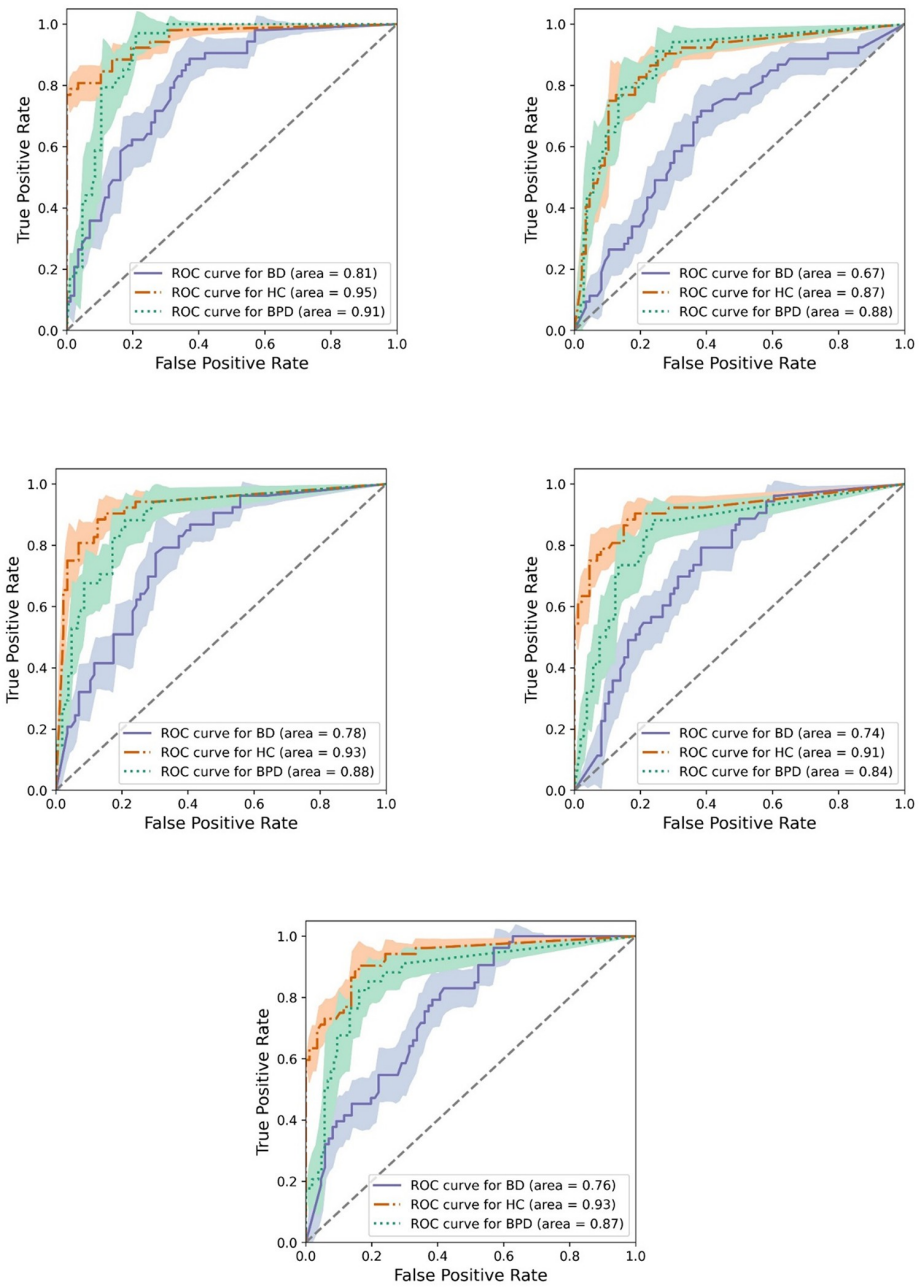
Receiver operating characteristic curves with 95% confidence interval for all models. Upper: MRSCM (left) and naive model (right); Middle: KNN (left) and PPCA (right); Lower: rfMICE.

The receiver operating characteristic curves for three groups from all models under hard voting were plotted with 95% confidence level in Fig 5 with areas under curve (auc) recorded in the brackets. AUC values were calculated in the one-vs-rest fashion. MRLSM had the best ability in identifying all diagnostic groups in terms of auc. Consistent with f1 scores in Table 4, all models had their lowest auc from ROC of bipolar group, which implies it is more likely for bipolar participants to be misplaced into the other two groups.

#### Further comparison

We examined the raw weekly data from participants who were recognised by MRLSM only. For this purpose we picked two participants as examples, one with high proportion of missing responses and another one with full record.

**Fig 6:**
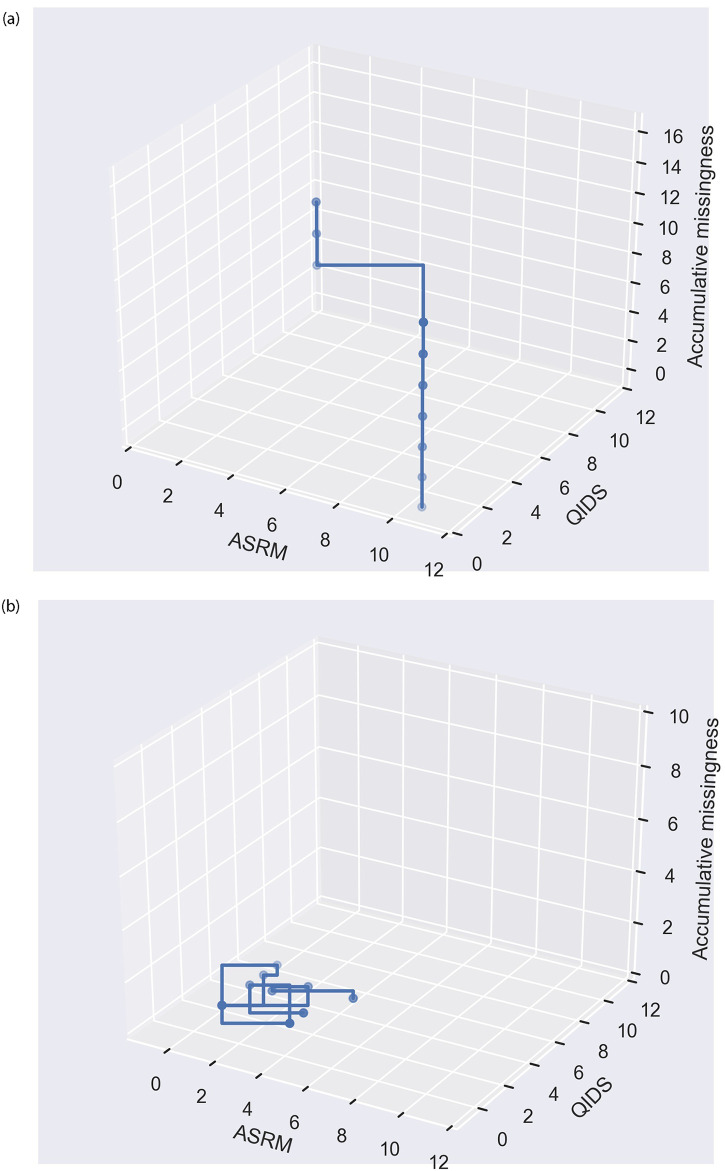
One participant who who did not miss a week. Randomly sampled ten-week data trajectory (weekly self-reported scores from ASRM, QIDS and missingness) of two participants who were recognised by MRLSM only. One participant missed over 70% weeks and another one did not miss a week. (a) One participant who missed over 70% weeks. (b) One participant who who did not miss a week.

The first example is a participant who missed over 70% weeks during their entire study. To be de-identifiable, Fig 6a shows weekly data of a randomly picked ten-week window, where one can observe three responses among the ten weeks. Given the high prevalence of missingness, we were not surprised that the imputation methods KNN, PPCA and rfRICE did not give reliable inference and thus led to wrong classification results. MRLSM on the other hand, treated missing values as a new signal, extracted a more faithful representation features and concluded a correct diagnosis.

The second example is a participant who did not miss a week during their participation in the study. In this case, no imputation is required and the naive model, which averages weekly scores, draw a wrong conclusion. Perhaps because this participant had comparably higher or lower average scores than other participants in the same diagnostic group. MRLSM recognised this participant. A significant part of the signature score came from the sudden mood changes, which you may observe from Fig 6b, even though this event occurred over short period of time.

#### Feature importance

The random forest algorithm we used presents a ranking of feature importance. We examined this ranking. The ten features of MRLSM ranked most significant are briefly summarised in Table 5. This ranking placed the accumulated incremental effects from scores of the four questionnaires and the missing signal as the most important. However, the higher-order interaction effects involving the missing signal also played an important role in decision making for classification.

**Table 5:** Feature importance of the MRLSM.

Rank	Importance	Feature interpretation
**1**	0.1506	Incremental effects of QIDS
**2**	0.1113	Incremental effects of GAD-7
**3**	0.0990	Incremental effects of EQ-5D
**4**	0.0512	Incremental effects of ASRM
**5**	0.0126	Incremental effects of the missing signal
**6**	0.0119	Interaction among EQ-5D, GAD-7 and the missing signal
**7**	0.0111	Interaction among QIDS, GAD-7 and the missing signal
**8**	0.0108	Interaction between QIDS and GAD-7
**9**	0.0108	Interaction between EQ-5D and the missing signal
**10**	0.0107	Interaction between GAD-7 and the missing signal

#### Spectrum analysis

**Fig 7:**
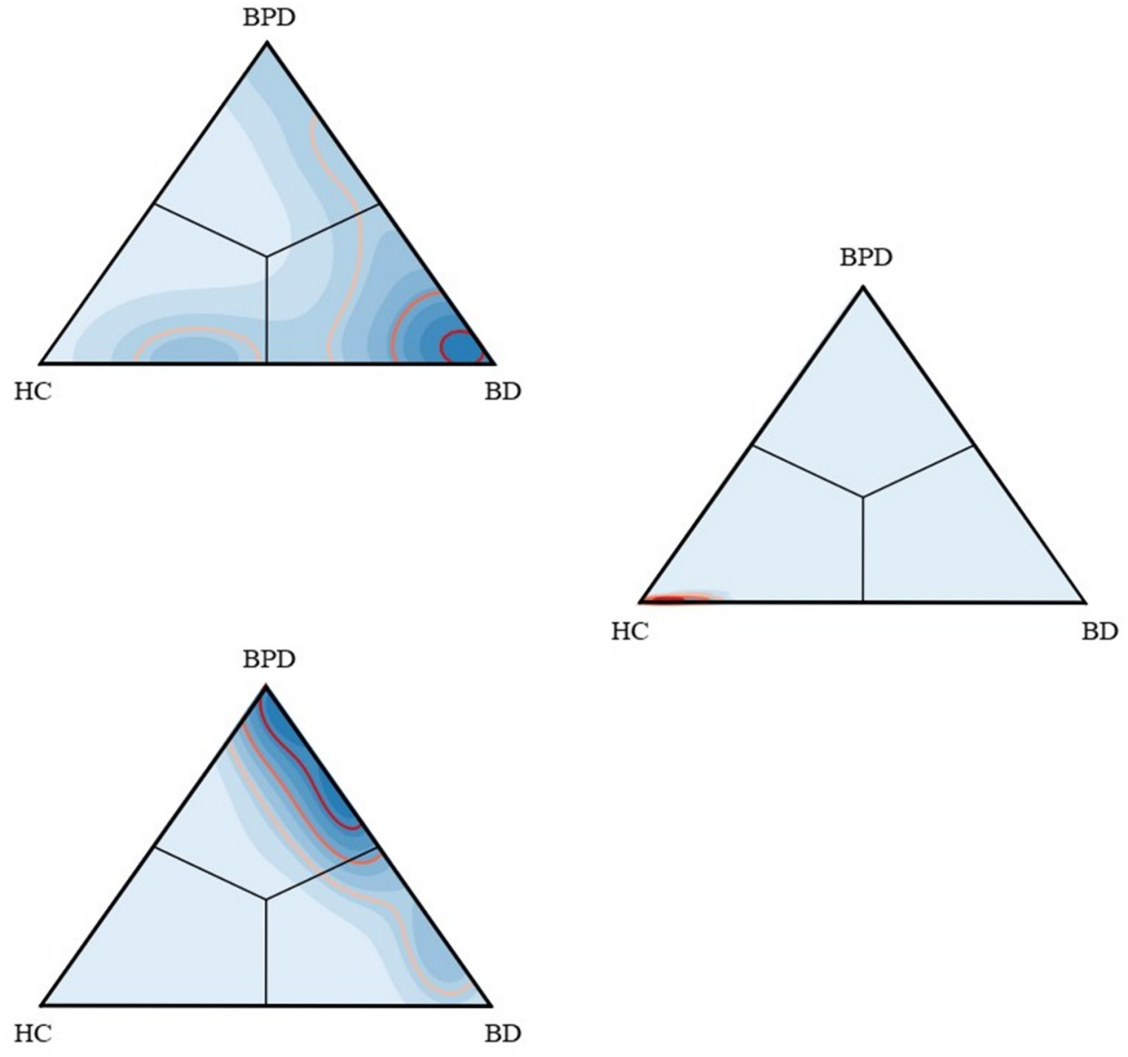
Density plots for the predicted diagnosis from MRLSM: Darker blue areas indicate higher density values, i.e., events that are more likely to happen, and vice versa; red lines indicate the 75% (the lightest red), 50%, 25% (the darkest red) boundaries of density contours, i.e., the events within the area enclosed by the 75% contour line is with probability 75% to happen. Upper: density plot of the predicted diagnosis for BD group. Middle: density plot for the predicted diagnosis for HC group. Lower: density plot for the predicted diagnosis for BPD group.

In Fig 7, the triangle spectrum of the predicted diagnosis from MRLSM are plotted. In each of the plots, the regions of highest density of participants are located in the correct corner of the triangle. The greatest consistency is with the healthy participants. Meanwhile, the probabilities of misdiagnosis to other groups can be measured by comparing the distances to the other two vertices to the distance to the right vertex. For instance, one can deduce from the middle subplot that the likelihood of misplacing healthy participants into the borderline group is very low. The lower subplot shows the other way around: BPD participants are unlikely to be misidentified as healthy control. The upper subplot shows that the bipolar participant can be misidentified as healthy control or BPD with similar probability.

## Discussion

This paper introduces the missing-response-incorporated log-signature random forest models and have them tested on the concatenated ASRM/QIDS/EQ-5D/GAD-7 data. The original database consists of longitudinal self-reported mood data. The participant was reminded to respond once a week, but could respond anytime they wished. The missing response is defined as having not reported their mood before the next reminder a week later. At least 25% of the participant enrolled weeks had a missing response (Fig 1). These missing response records are informative and in our view they should be ignored. By integrating the missing response records into the multimodal stream as an extra coordinate, and using a genuinely multimodal data analysis, it is straightforward to extract exact amount of additional information allowing better discrimination between the diagnostic classes (ie, bipolar disorder, healthy control and borderline personality disorder). Note that the overall strategy for dealing with missing data we presented is not specific to this psychiatric context but does rely on having a flexible and robust approach to analysing multimodal and irregularly arriving data.

Our approach to analysing the irregular multimodal data is effective and has been successfully used in the range of different applications over the last couple of years. Signature-based methods were adopted by Perez et al. [1] and outperformed neuroimaging [34] and verbal fluency [35]. We focus on differentiating between the three diagnostic classes and demonstrate that the missing-response-incorporated log-signature-based model is superior to a commonly used metric (the naive model) and to various imputation models. Our result outperforms the approach in [1] because we take account of the information contained in the missing data. It is interesting to compare Fig 3 in [1] and Fig 7, the classifications are significantly tighter (more localised). In addition, a bipolar diagnosis can be confused with a healthy participant or a borderline personality participant, but there are almost no cases where an individual with the bipolar diagnosis might be scored equally as a healthy and a borderline personality participant. Without the missing data information, this case occurred more frequently in the previous analysis (cf Fig 3 in [1]).

For most models, the performance of diagnostic group classification (Table 4 and Fig 5) for BD participants was the worst among the three groups, partly due to their greater range of mood states compared with BPD and partly due to their sparser trajectories compared with HC. The corresponding f1 score for classification using naive model was just above 0.5. The poor performance alerted the unreliability of this commonly used metric in identifying BD participants when missingness commonly exists. On the other hand, by incorporating extra valuable information like missing responses into features, the log-signature-based model lifted the f1 score for identifying BD participants to above 0.65 and for BPD participants to around 0.75, with less than one fourth BD (resp. BPD) participants being misclassified as BPD (resp. BD). Compared to KNN, the best model of all imputation methods, MRLSM showed its significant advantage in recognising BPD and BD, both groups having high proportion of missing data. This demonstrates the ability of the missing-response-incorporated log-signature features to capture and learn the inherent differences in patterns of mood and missingness between BPD and BD.

The good performance of all models in identifying HC is a consequence of much lower prevalence of missing responses compared to other two groups (Figs 1 and 2). Under such condition, imputation methods were able to draw reasonable inference based on adequate available information and MRLSM was still superior to the rest models due to its ability of capturing the intrinsic patterns and trends of the data streams and giving faithful representation features. Note that its advantage in f1 scores to KNN was reduced from 5.0% (BD) and 6.3% (BPD) to 1.9% (HC). This implies that the signature approach is more applicable and favourable when there is more missing data.

By treating missing responses as a signal, the proposed signature approach makes the previously hidden information visible and the superiority of the signature approach in turn highlights that missing data conveys valuable clinical information. This was also supported by the example shown in Fig 6a and feature importance of features involving the missing signal in Table 5. Note that the top four features in Table 5 have the same effect as the average scores from naive model. This is because the incremental effect of a score trajectory can be treated as the difference between the accumulated score in the initial week and the accumulated score in the ending week, where the latter one amounts to a multiple of the average score over the period. Equivalently, the features used in the naive model (and any other models) can be recovered by the (log-)signature features based on the fact that any functionals on the path can be rewritten as a function on the signature. This implies that the signature approach outperformed the naive model due to its correctly extracting useful information hidden in the missing signal.

Spectrum analysis showed the clear separation between BPD and HC in Fig 7 (the middle and bottom subplots). As a consequence, we had the ‘V’ shape in the top subplot, and the overlap between BD and HC groups and the one between BD and BPD groups were resulted from different causes. The former overlap is consistent with the analysis in [1] and with clinical experience. While BD is defined by episodes of elated and depressed mood it is also associated with periods of stable mood. It is also likely that monitoring of mood enables people to better understand their condition and proactively take steps to prevent subsequent mood episode. For both of these reasons an overlap with HC participants is to be expected. Similarly for the latter overlapping, when one BD participant suffered from depression and mood instability during their entire study, the corresponding data is much like the data patterns given by most BPDs and leads to a wrong classification. These effects both suggest that for some participant, their study length may not be long enough for a conclusion, or that the diagnosis was wrong or had changed. However, we found a much clearer differentiation between diagnostic groups than previous work [1] suggesting that the inclusion of missing data added useful information.

Compared to the middle subplot of Fig 7, the overlap between BPD and BD in the bottom one is significant. With the lowest participant number, BPD therefore had the fewest features for the classification task, which in turn leveraged misclassification.

### Limitations and implications

The missing-response-incorporated signature-based features offer a systematic approach to the analysis of longitudinal self-reported mood data with the presence of non-randomly distributed missing values. It can be easily utilised with various machine learning methods for learning tasks on other databases containing missing information. The reasons for the moderate accuracies using MRLSF are three-fold: the full potential of signature features is hindered by the small and unblanced dataset, the proposed feature extraction method might not be the optimal, and the concatenated mood data was analysed on the overall-score level instead of on the question-score level. In the future, we would prioritise on two explorations: assessing our proposed method on different mental health datasets, and adjusting MRLSF to the “optimal” signature-based feature by adding reasonable metrics/transformations which account for different attributes.
